# CD47 Expression in Non-Melanoma Skin Cancers and Its Clinicopathological Implications

**DOI:** 10.3390/diagnostics12081859

**Published:** 2022-07-31

**Authors:** Seongsik Bang, Seungyun Jee, Hwangkyu Son, Hyebin Cha, Hosub Park, Jaekyung Myung, Joo Yeon Ko, Hyunsung Kim, Seungsam Paik

**Affiliations:** 1 Department of Pathology, Seoul Hospital, Hanyang University College of Medicine, Seoul 04763, Korea; grypony@naver.com (S.B.); jee.seung.yun@gmail.com (S.J.); ganzi4900@gmail.com (H.S.); chbin0111@gmail.com (H.C.); parkhstm@gmail.com (H.P.); tontos016@naver.com (J.M.); 2 Department of Dermatology, Seoul Hospital, Hanyang University College of Medicine, Seoul 04763, Korea; drko0303@ehanyang.ac.kr

**Keywords:** non-melanoma skin cancer, basal cell carcinoma, squamous cell carcinoma, CD47, immunohistochemistry

## Abstract

CD47 is a cell surface molecule and regulates diverse cellular responses. CD47 is highly expressed in cancer cells and has potential as a therapeutic target and prognostic factor in cancer patients. The expression patterns of CD47 in basal cell carcinoma (BCC), squamous cell carcinoma (SCC) and its precursor lesions, and its clinicopathological significance were investigated. CD47 expression was evaluated by immunohistochemistry in 152 cases of BCC and 71 cases of SCC. For comparison of CD47 expression, actinic keratosis (AK), squamous cell carcinoma in situ (SCCIS), keratoacanthoma (KA), and normal skin (NS) tissue were used. CD47 expression in BCC was significantly lower than that of SCC (*p* < 0.001). CD47 expression levels in SCC and KA were significantly higher than those of NS and AK (*p* < 0.05). High CD47 expression was significantly associated with the presence of ulceration (*p* = 0.005) and a deeper level of invasion (*p* = 0.011) in BCC. In addition, high CD47 expression was significantly associated with the presence of ulceration (*p* = 0.019) and larger tumor size (*p* = 0.004) in SCC. CD47 expression was associated with tumorigenesis and tumor progression in non-melanoma skin cancers.

## 1. Introduction

Non-melanoma skin cancer (NMSC) is the most commonly diagnosed cancer worldwide [1]. Although cancer-related deaths from NMSC are very low, the incidence rate is increasing thereby making it an important healthcare issue [2,3]. With few exceptions, the majority of NMSC cases are basal cell carcinoma (BCC) and squamous cell carcinoma (SCC) originating from epidermal keratinocytes. BCC and SCC are commonly associated with risk factors, such as cumulative exposure to ultraviolet (UV) radiation and Fitzpatrick skin types I and II [4,5]. However, there are significant differences in etiology, molecular genetics, and prognosis of these two types of cancer [5,6].

Cluster of differentiation 47 (CD47, IAP) is a member of the immunoglobulin superfamily and is involved in diverse cellular responses by interaction with specific ligands, such as signal regulatory protein α (SIRPα) [7]. The CD47-SIRPα signaling pathway is relatively well known to prevent phagocytosis of macrophages [8]. In many studies, human cancer cells showed high CD47 expression and inhibition of the CD47-SIRPα signaling pathway using anti-CD47 antibodies promoted the removal of cancer cells. As a result, CD47 is considered a potential therapeutic target [9,10,11]. In addition, high CD47 expression is usually associated with poor prognosis in cancer patients, thus showing potential as a prognostic marker [12].

For NMSC, Akel et al. reported on CD47 expression in cutaneous SCC and its precursors. They identified that CD47 expression was significantly higher in squamous cell carcinoma in situ (SCCIS) compared to actinic keratosis (AK), and in SCC compared to SCCIS. They suggested that a gradual increase in CD47 expression is associated with tumor progression [13]. However, CD47 expression in BCC has never been reported and the clinicopathological significance of CD47 expression in NMSC has never been studied.

In this study, we investigated CD47 expression in BCC, SCC, and its precursor lesions using immunohistochemistry (IHC). In addition, we analyzed the association between clinicopathological parameters and CD47 expression in BCC and SCC.

## 2. Materials and Methods

### 2.1. Clinical Data Collection and Pathological Evaluation

Patients diagnosed with BCC or SCC at Hanyang University Hospital (Seoul, Korea) were enrolled in the study. To assess the correlations between CD47 expression and uncommon features in NMSC (local recurrence, perineural invasion, and metastasis), we included the maximum number of cases that were collectible for a limited period (September 2008 and June 2019). In all, 172 cases of BCC and 74 cases of SCC were retrospectively reviewed, of which 20 cases of BCC and three cases of SCC were excluded due to unavailable tumor tissue. An additional 145, 73, 19, and 20 samples of AK, SCCIS, keratoacanthoma (KA), and normal skin (NS) were obtained to compare CD47 expression with NMSC, respectively. Medical records were reviewed to obtain the clinical characteristics, including patient age, sex, tumor location, ulceration, local recurrence, and lymph node metastasis. A review of tissue slides and pathologic reports was performed by two pathologists (S.B. and S.P.). Tumor size, histological subtypes of BCC, histological grade of SCC, level of invasion, and perineural invasion were reviewed. Among the histological subtypes of BCC, micronodular, infiltrating, sclerosing/morphoeic, and basosquamous were considered aggressive growth patterns [14,15]. The histologic grade of SCC was divided using a three-level grading system (G1: well-differentiated, G2: moderately differentiated, and G3: poorly differentiated) according to the proportion of keratinizing tumor cells [16]. The basic characteristics of BCC cases and SCC cases are summarized in Supplementary Tables S1 and S2, respectively.

### 2.2. Tissue Microarray Construction and Immunohistochemistry

We constructed tissue microarrays (TMA) to efficiently evaluate biomarker expression [17]. Manual microarray systems (Tissue Microarray Set, Labro, Seoul, Korea) and formalin-fixed paraffin-embedded tissues were used for tissue microarray (TMA) construction. We selected a representative area of the tumor by light microscopy and collected tissue cores (3.0 mm) from the corresponding donor block. Then, the tissue cores were transferred to a recipient block consisting of 6 × 5 samples. Each TMA block was cut into 4-μm-thick sections and stored at −70 to −80 °C.

IHC was used to evaluate CD47 expression in tissue samples of produced TMA. According to the manufacturer’s protocol, we performed IHC with the Benchmark XT automated staining system (Ventana Medical Systems, Tucson, AZ, USA), and a recombinant anti-CD47 antibody (EPR21794, Abcam, Cambridge, UK; diluted 1:200) was used as a primary antibody.

### 2.3. Interpretation of Immunohistochemistry

**Figure 1:**
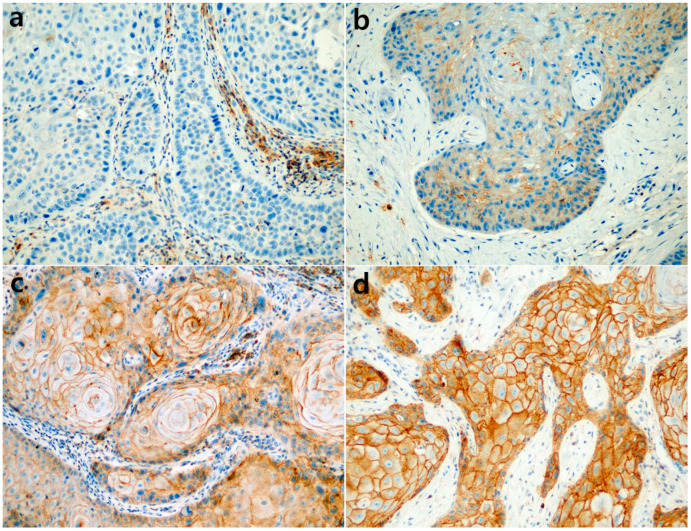
Representative photomicroscopic images of CD47 expression in squamous cell carcinoma. The staining intensity was classified as: (**a**) negative, 0; (**b**) weak, 1; (**c**) moderate, 2; and (**d**) strong, 3 ((**a**–**d**), 200×).

CD47 expression was evaluated by two pathologists (S.B. and S.P.) without access to clinicopathological data. The membranous staining of the tumor cells was considered to be positive, and the histoscore (H-score) method was used for semi-quantitative evaluation as previously described by Arrieta et al. [18]. The H-score was calculated as the product of the staining intensity and percentage of positive cells, ranging from 0 to 300 for each case. The staining intensity was classified as 0 to 3 (0: negative, 1: weak, 2: moderate, and 3: strong). Representative images according to the intensity are presented in Figure 1. A receiver operating characteristics (ROC) curve analysis was then used to determine the optimal cutoff value for high CD47 expression in BCC and SCC cases, respectively.

### 2.4. Statistical Analyses

The Kruskal–Wallis test was performed to compare CD47 expression in BCC, SCC, AK, SCCIS, KA, and normal skin tissue. The Mann–Whitney U-test was used as a post hoc analysis to evaluate the mean difference between groups. Pearson’s chi-square (χ2) and Fisher’s exact tests were performed to evaluate the association between CD47 expression and clinicopathological characteristics in SCC and BCC. SPSS software version 25.0 (IBM, Armonk, NY, USA) was used for all statistical analyses, and a two-tailed *p*-value < 0.05 was regarded as statistically significant.

## 3. Results

### 3.1. CD47 Expression in Skin Tumors and Normal Tissue

**Figure 2:**
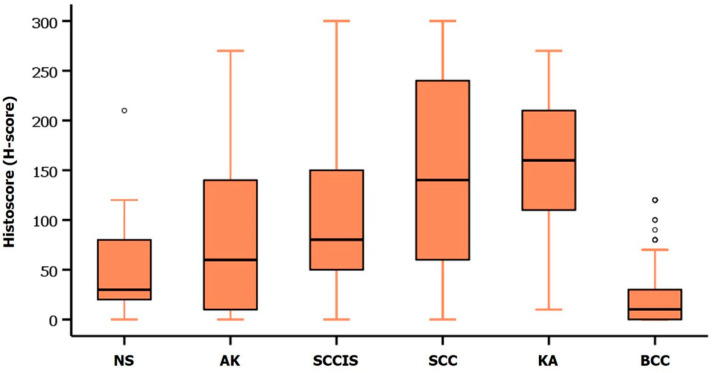
Comparison of CD47 expression in normal skin (NS), actinic keratosis (AK), squamous cell carcinoma in situ (SCCIS), squamous cell carcinoma (SCC), keratoacanthoma (KA), and basal cell carcinoma (BCC). (*p* < 0.001, Kruskal–Wallis test). Circles represent mild outliers.

**Figure 3:**
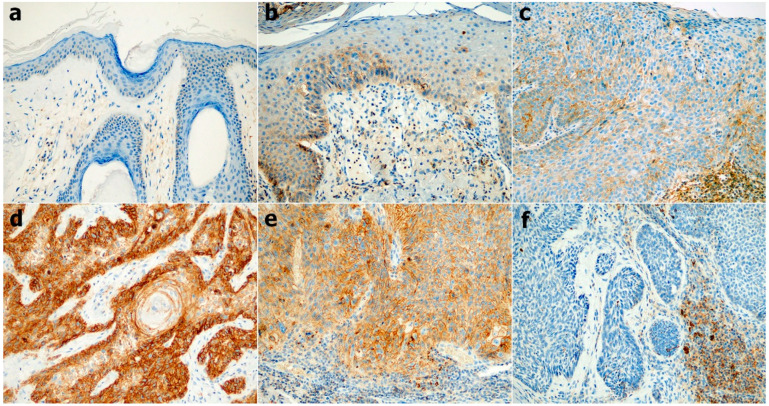
CD47 expression in normal skin (**a**), actinic keratosis (**b**), squamous cell carcinoma in situ (**c**), squamous cell carcinoma (**d**), keratoacanthoma (**e**), and basal cell carcinoma (**f**) ((**a**–**f**), 200×).

We evaluated CD47 expression in NMSC (BCC and SCC), AK, SCCIS, KA, and NS, and then calculated a H-score of CD47 expression in each group. The CD47 expression showed significant differences in each group (*p* < 0.001, Kruskal–Wallis test, Figure 2), and representative images of CD47 expression are shown in Figure 3. By post hoc analyses, the median H-score of CD47 expression in BCC was found to be significantly lower than those of AK, SCCIS, SCC, and KA (*p* < 0.001). The median H-score of CD47 expression in SCC and KA was significantly higher than those of NS and AK (*p* < 0.05). The median H-score of CD47 expression between NS, AK, and SCCIS gradually increased, however, there was no statistically significant difference.

### 3.2. Correlations between CD47 Expression and Clinicopathological Parameters in BCC

By ROC curve analysis, we divided BCC cases into low and high expression groups (H-score ≤ 20 and H-score > 20, respectively). A total of 45 out of 152 BCC cases (29.6%) were included in the high CD47 expression group. High CD47 expression showed a significant association with the presence of ulceration (*p* = 0.005) and deeper level of invasion (*p* = 0.011). Patient’s age, sex, location, local recurrence, tumor size, histological subtypes, and perineural invasion were not significantly associated with CD47 expression. The correlations between CD47 expression and clinicopathological characteristics in BCC cases are summarized in Table 1.

### 3.3. Correlations between CD47 Expression and Clinicopathological Parameters in SCC

By ROC curve analysis, we divided SCC cases into low and high expression groups (H-score ≤ 160 and H-score > 160, respectively). Of 71 SCC cases, 29 (40.8%) were included in the high CD47 expression group. High CD47 expression showed a significant association with the presence of ulceration (*p* = 0.019) and larger tumor size (*p* = 0.004). Patient’s age, sex, location, local recurrence, lymph node metastasis, histological grade, level of invasion, and perineural invasion were not significantly associated with CD47 expression. The correlations between CD47 expression and clinicopathological characteristics in SCC cases are summarized in Table 2.

## 4. Discussion

In this study, we found that there was a significant difference in CD47 expression in BCC and SCC. CD47 expression in BCC was significantly lower than that of SCC (*p* < 0.001). CD47 expression in SCC and KA was significantly higher than those of NS and AK (*p* < 0.05). High CD47 expression was significantly associated with the presence of ulceration (*p* = 0.005) and a deeper level of invasion (*p* = 0.011) in BCC. High CD47 expression was significantly associated with the presence of ulceration (*p* = 0.019) and larger tumor size (*p* = 0.004) in SCC.

Distinguishing between SCC and BCC by showing typical histological characteristics is usually not a challenging task. However, in cases of SCC showing basaloid features or BCC showing squamous differentiation, it may be problematic to differentiate them. Therefore, there have been many studies on diagnostic markers for distinguishing between BCC and SCC. IHC is a useful method for differential diagnosis of epithelial skin tumors in daily practice, and immunopanel composed of several IHC markers can be helpful when the histological characteristics of SCC and BCC overlap [19]. As promising candidates, Ber-EP4, EMA, CAM5.2, androgen receptor, p16, SOX2, MOC-31, etc., can be considered [20,21,22]. In this study, BCC showed significantly lower CD47 expression compared to SCC, showing potential as a diagnostic tool.

Increased CD47 expression in SCC has been reported in several studies. Suzuki et al. found that CD47 expression was higher in esophageal SCC samples compared to adjacent non-cancerous tissues [23]. Yang et al. identified significantly higher mRNA and protein expression of CD47 in laryngeal SCC compared to para-carcinoma tissues [24]. Ye et al. and Wu et al. compared CD47 expression levels in normal mucosa, dysplastic lesion, and head and neck SCC, and reported a gradual increase in CD47 expression from normal tissue to cancer [25,26]. Akel et al. reported a gradual increase in CD47 expression along the spectrum from AK to SCCIS to cutaneous SCC [13]. Similar to these results, we found high CD47 expression in SCC and KA compared to normal skin and AK, suggesting that CD47 expression is related to tumorigenesis of cutaneous squamous lesions.

CD47 is known to promote the growth, invasion, and migration of cancer cells. Willingham et al. showed that anti-CD47 antibody inhibited tumor growth and inhibited metastasis using xenotransplantation models [27]. Wang et al. showed that silencing of CD47 through siRNA inhibited melanoma growth and lung metastasis [28]. Zhao et al. reported that downregulation of CD47 inhibited tumor growth, cell invasion, and metastasis in non-small cell lung cancer [29]. Wang et al. reported that CD47 overexpression in ovarian cancer cell lines promoted cancer cell growth and motility [30]. In our study, we analyzed the association between CD47 expression and clinicopathological parameters and identified that high CD47 expression was associated with deeper level of invasion in BCC and larger tumor size in SCC. In addition, KA, which is characterized by rapid tumor growth till 1–2 cm [31], showed higher CD47 expression compared to NS and AK.

The molecular mechanisms regulating CD47 expression in tumors are not still unclear. Recently, two studies have been performed using next-generation sequencing (NGS) to investigate the association between CD47 expression and genetic alterations, or CD47 expression and other gene expressions. Cho et al. found that 18q21 gain/amplification was associated with high CD47 expression in diffuse large B-cell lymphoma [32]. Tseng et al. revealed that heat shock protein family A member 5 (HSPA5) had a positive correlation with CD47 expression in Oral SCC cell lines using bioinformatic analysis [33]. There are no molecular biological studies of CD47 expression in NMSCs, and further studies are needed.

In conclusion, we evaluated the CD47 expression in NMSC by immunohistochemistry and identified its potential as a diagnostic tool to differentiate between BCC and SCC. In addition, we found that CD47 expression was associated with tumorigenesis and tumor progression, suggesting its potential as a prognostic biomarker.

## Figures and Tables

**Table 1:** Correlations between CD47 expression and clinicopathological characteristics in cases with basal cell carcinoma (*n* = 152).

Variables	CD47 Expression		*p*-Value
	Low Expression (%) (*n* = 107)	High Expression (%) (*n* = 45)	
Age			0.243
<70 years	46 (65.7%)	24 (34.3%)	
≥70 years	61 (74.4%)	21 (25.6%)	
Sex			0.627
Female	64 (71.9%)	25 (28.1%)	
Male	43 (68.3%)	20 (31.7%)	
Location			0.275 ^a^
Sun-protected skin	15 (83.3%)	3 (16.7%)	
Sun-damaged skin	92 (68.7%)	42 (31.3%)	
Ulceration			0.005
No	100 (74.1%)	35 (25.9%)	
Yes	7 (41.2%)	10 (58.8%)	
Local recurrence			1.000 ^a^
No	105 (70.5%)	44 (29.5%)	
Yes	2 (66.7%)	1 (33.3%)	
Tumor size ^b^			1.000 ^a^
<2.0 cm	72 (67.3%)	35 (32.7%)	
≥2.0 cm	8 (66.7%)	4 (33.3%)	
Histological subtypes ^c^			0.649
Less aggressive and others	78 (72.2%)	30 (27.8%)	
Aggressive	29 (65.9%)	15 (34.1%)	
Level of invasion			0.011
Limited to the dermis	89 (75.4%)	29 (24.6%)	
Deeper than the dermis	18 (52.9%)	16 (47.1%)	
Perineural invasion			0.582 ^a^
Not identified	105 (70.9%)	70 (29.1%)	
Present	2 (50.0%)	2 (50.0%)	

^a^ Fisher’s exact test. ^b^ Tumor size, 33 cases missed. ^c^ Less aggressive and others: nodular, superficial, fibroepithelial, and BCC with adnexal differentiation; Aggressive: micronodular, infiltrating, sclerosing/morphoeic, and basosquamous.

**Table 2:** Correlations between CD47 expression and clinicopathological characteristics in cases with squamous cell carcinoma (*n* = 71).

Variables	CD47 Expression		*p*-Value
	Low Expression (%) (*n* = 42)	High Expression (%) (*n* = 29)	
Age			0.593
<70 years	11 (64.7%)	6 (35.3%)	
≥70 years	31 (57.4%)	23 (42.6%)	
Sex			0.734
Female	20 (57.1%)	15 (42.9%)	
Male	22 (61.1%)	14 (38.9%)	
Location			0.298 ^a^
Sun-protected skin	4 (40.0%)	6 (60.0%)	
Sun-damaged skin	38 (62.3%)	23 (37.7%)	
Ulceration			0.019
No	34 (68.0%)	16 (32.0%)	
Yes	8 (38.1%)	13 (61.9%)	
Local recurrence			1.000 ^a^
No	38 (58.5%)	27 (41.5%)	
Yes	4 (66.7%)	2 (33.3%)	
Lymph node metastasis			0.298 ^a^
No	41 (61.2%)	26 (39.8%)	
Yes	1 (25.0%)	3 (75.0%)	
Tumor size ^b^			0.004 ^a^
<2.0 cm	27 (65.9%)	14 (34.1%)	
≥2.0 cm	4 (23.5%)	13 (76.5%)	
Histological grade			0.129
G1	33 (64.7%)	18 (35.3%)	
G2 or G3	9 (45.0%)	11 (55.0%)	
Level of invasion			0.200
Limited to the dermis	32 (64.0%)	18 (36.0%)	
Deeper than the dermis	10 (47.6%)	11 (52.4%)	
Perineural invasion			0.298 ^a^
Not identified	41 (61.2%)	26 (38.8%)	
Present	1 (25.0%)	3 (75.0%)	

^a^ Fisher’s exact test. ^b^ Tumor size, 13 cases missed.

## Data Availability

Not applicable.

## Supplementary Materials

The following supporting information can be downloaded at: https://www.mdpi.com/article/10.3390/diagnostics12081859/s1, Table S1: Baseline characteristics of cases with basal cell carcinoma (*n* = 152); Table S2: Baseline characteristics of cases with squamous cell carcinoma (*n* = 71).
