# Supplementary material for: CD47 Expression in Non-Melanoma Skin Cancers and Its Clinicopathological Implications
Source: Diagnostics (Basel). 2022 Jul 31;12(8):1859. doi: 10.3390/diagnostics12081859 (PMC9406543; doi:10.3390/diagnostics12081859)
Supplement: Supplementary file 1 [file diagnostics-12-01859-s001.zip › diagnostics-1823395-supplementary.pdf]

Table S1. Baseline characteristics of cases with basal cell carcinoma (n = 152).

Baseline characteristics	Case No. (%)
Age, median (range, year)	68 (29-98)
Sex	
Female	89 (58.6%)
Male	63 (41.4%)
Location	
Scalp	7 (4.6%)
Face	122 (80.3%)
Neck	3 (2.0%)
Upper extremity	1 (0.7%)
Lower extremity	3 (2.0%)
Trunk	11 (7.2%)
Urogenital region	5 (3.3%)
Ulceration	
No	135 (88.8%)
Yes	17 (11.2%)
Local recurrence	
No	149 (98.0%)
Yes	3 (2.0%)
Tumor size, mean (range, cm)	1.1 (0.25-5.0)
Histological subtypes	
Superficial	10 (6.6%)
Nodular	91 (59.9%)
Micronodular	9 (5.9%)
Infiltrating	2 (1.3%)
Sclerosing/morphoeic	1 (0.7%)
Basosquamous	2 (1.3%)
Fibroepithelial	1 (0.7%)
Basal cell carcinoma with adnexal differentiation	4 (2.6%)
Mixed	32 (21.1%)
Level of invasion	
Papillary dermis	11 (7.2%)
Reticular dermis	107 (70.4%)
Subcutis	22 (14.5%)
Skeletal muscle	12 (7.9%)
Perineural invasion	
Not identified	148 (97.4%)
Present	4 (2.6%)

Table S2. Baseline characteristics of cases with squamous cell carcinoma (n = 71).

Baseline characteristics	Case No. (%)
Age, median (range, year)	75 (33-96)
Sex	
Female	35 (49.3%)
Male	36 (50.7%)
Location	
Scalp	7 (9.9%)
Face	38 (53.5%)
Neck	2 (2.8%)
Upper extremity	7 (9.9%)
Lower extremity	12 (16.9%)
Trunk	5 (7.0%)
Ulceration	
No	50 (70.4%)
Yes	21 (29.6%)
Local recurrence	
No	65 (91.0%)
Yes	6 (8.5%)
Lymph node metastasis	
No	67 (94.4%)
Yes	4 (5.6%)
Tumor size, mean (range, cm)	2.5 (0.3-14.0)
Histological grade	
Well differentiated (G1)	51 (71.8%)
Moderately differentiated (G2)	17 (23.9%)
Poorly differentiated (G3)	3 (4.2%)
Level of invasion	
Papillary dermis	14 (19.7%)
Reticular dermis	36 (50.7%)
Subcutis	14 (19.7%)
Skeletal muscle or bone	7 (9.9%)
Perineural invasion	
Not identified	67 (94.4%)
Present	4 (5.6%)
